# Approaches for assessing performance of high-resolution mass spectrometry–based non-targeted analysis methods

**DOI:** 10.1007/s00216-022-04203-3

**Published:** 2022-07-07

**Authors:** Christine M. Fisher, Katherine T. Peter, Seth R. Newton, Andrew J. Schaub, Jon R. Sobus

**Affiliations:** 1 Center for Food Safety and Applied Nutrition, US Food and Drug Administration, College Park, MD 20740 USA; 2 Center for Urban Waters, University of Washington Tacoma, Tacoma, WA 98421 USA; 3 Former: National Institute of Standards and Technology, Charleston, SC 29412 USA; 4 US Environmental Protection Agency, 109 TW Alexander Dr., Research Triangle Park, NC 27711 USA; 5 Intelligent Systems Division, Southwest Research Institute, San Antonio, TX 78228 USA

**Keywords:** HRMS, NTA, Sample classification, Chemical identification, Chemical quantitation, Performance assessment

## Abstract

Non-targeted analysis (NTA) using high-resolution mass spectrometry has enabled the detection and identification of unknown and unexpected compounds of interest in a wide range of sample matrices. Despite these benefits of NTA methods, standardized procedures do not yet exist for assessing performance, limiting stakeholders’ abilities to suitably interpret and utilize NTA results. Herein, we first summarize existing performance assessment metrics for targeted analyses to provide context and clarify terminology that may be shared between targeted and NTA methods (e.g., terms such as accuracy, precision, sensitivity, and selectivity). We then discuss promising approaches for assessing NTA method performance, listing strengths and key caveats for each approach, and highlighting areas in need of further development. To structure the discussion, we define three types of NTA study objectives: sample classification, chemical identification, and chemical quantitation. Qualitative study performance (i.e., focusing on sample classification and/or chemical identification) can be assessed using the traditional confusion matrix, with some challenges and limitations. Quantitative study performance can be assessed using estimation procedures developed for targeted methods with consideration for additional sources of uncontrolled experimental error. This article is intended to stimulate discussion and further efforts to develop and improve procedures for assessing NTA method performance. Ultimately, improved performance assessments will enable accurate communication and effective utilization of NTA results by stakeholders.

**Graphical abstract:**

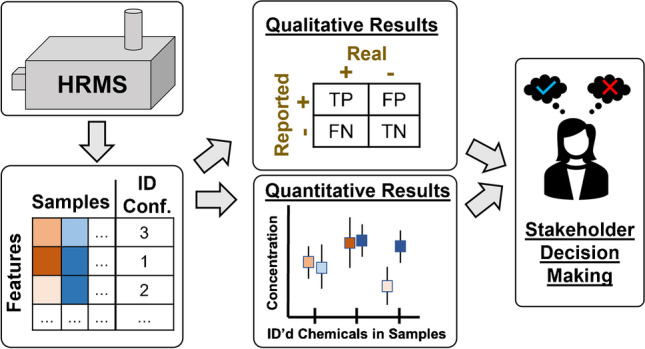

**Supplementary Information:**

The online version contains supplementary material available at 10.1007/s00216-022-04203-3.

## Introduction

Targeted analytical chemistry methods are foundational to safety assessment and management activities in a variety of fields. These methods provide critical quantitative measurement data for specific, known chemicals that support a variety of activities, including exposure assessment, hazard identification, dose–response evaluation, and eventual risk characterization. Targeted method performance criteria (i.e., thresholds for selectivity, sensitivity, accuracy, and precision) are well-defined, widely accepted, and adaptable to any given application, facilitating communication about results between analysts and stakeholders (i.e., any individuals with an interest in the data itself or data-driven decisions) [1–3]. Yet, in recent years, management agencies and supporting laboratories have been increasingly tasked with identifying new, unknown, or unexpected chemical stressors in complex samples (notable examples include novel per- and poly-fluorinated alkyl substances [PFAS] in soil [4] and water [5, 6], biomarkers of exposure to burn pits in serum [7], and discharged pharmaceuticals in wastewater [8]). Furthermore, researchers now routinely face the challenge of characterizing large classes of diverse chemicals (e.g., PFAS [9], illicit designer drugs [10, 11] and steroids [12], and chemical warfare agents [13]) that may impact humans and/or ecological species. Existing targeted methods are simply not well suited to address these types of environmental and public health challenges.

Non-targeted analysis (NTA) methods, often using high-resolution mass spectrometry (HRMS), have rapidly emerged to fill critical data gaps and address challenges not easily solved by targeted analyses. These methods are highly versatile and capable of rapidly detecting known compounds (without the need for preliminary experimentation to develop and optimize targeted methods for specific analytes) [14, 15], confidently identifying unknown/unexpected contaminants [5, 16], retrospectively assessing past exposures (via analysis of archived samples or data) [17, 18], and efficiently classifying samples based on data patterns [19, 20]. Moreover, NTA methods are amenable to virtually any sample medium, including air [21], water [5, 6, 14], sediment [22], soil [4, 23], dust [24], food [25, 26], consumer products [27], and biological specimens [7, 28]. NTA research tools and approaches are being developed and applied at a staggering rate. Yet, to date, most analytical laboratories have adopted NTA methods and results primarily within research (non-regulatory) applications. Furthermore, individual laboratories have developed their own criteria and procedures for determining that their methods are fit for specific purposes. However, a primary barrier to broader adoption of NTA data is the current lack of unified capabilities and criteria for effectively assessing and communicating NTA method performance [17, 29].

In contrast with targeted analytical methods, defining performance metrics for NTA methods remains challenging, limiting research transparency and stakeholder use of NTA data. Even within the NTA research community, there are not yet universal methods to describe the scope, certainty, and overall performance of any given NTA study. A standardized system of performance evaluation (eventually including third-party accreditation, echoing the criteria applied to targeted analyses) may therefore be needed before NTA can be incorporated into existing chemical monitoring and assessment frameworks. As momentum builds to directly integrate NTA into decision-making processes, so too does the need to establish uniform approaches for quantifying and communicating NTA method performance.

To address this challenge and stimulate further discussion, we herein discuss typical NTA study objectives, related performance metrics, and lingering issues that require community-wide discussion and eventual consensus. We focus on three NTA study objectives that can be used to categorize most NTA projects and that yield results most useful for stakeholders: sample classification, chemical identification, and chemical quantitation. We provide examples and recommendations regarding use of the proposed performance metrics and discuss potential biases and challenges. We note that our focus herein is on performance assessment approaches that provide an overall evaluation of the NTA method, rather than on the quality assurance/quality control (QA/QC) approaches that should be incorporated throughout any NTA workflow to evaluate specific method steps [30–35]. This article is intended to initiate the extremely challenging (but entirely necessary) discussion about remaining needs to achieve broader acceptance of NTA in the eyes of key stakeholders. Adoption of terminology and metrics described herein will help accelerate and broaden the utilization of NTA results by stakeholders such as analytical chemists, public health officials, and decision-makers.

## Targeted analysis objectives and performance assessment

Before considering NTA uses, metrics, and challenges, we briefly review targeted analysis objectives and performance criteria. We note that the performance metrics discussed in this section are not exhaustive and many institutions have specific guidelines for performance assessments of targeted analysis. However, a brief discussion of commonly used metrics is included to provide the necessary context for NTA practitioners, novices, and other stakeholders alike to better understand important differences between targeted analysis and NTA. Targeted analyses aim to quantitatively characterize pre-selected analytes in defined study samples. Low-resolution mass spectrometry (MS) or tandem mass spectrometry (MS/MS) is widely considered the gold-standard analytical platform for small molecule quantitation, although recent efforts toward use of high-resolution mass spectrometry are promising [36, 37] (for additional detail, see Electronic Supplementary Material (ESM) Text S1). The quality of targeted, quantitative analytical results can be evaluated using well-established performance metrics for ***selectivity***, ***sensitivity***, ***accuracy***, and ***precision*** (described below). These four criteria provide a foundation for optimizing and assessing the performance of any targeted analytical method, and in turn, deeming a method fit-for-purpose to provide credible data for specific chemicals, where the results are considered unambiguously true within clearly defined tolerances. To contrast the use of these terms in NTA (see section “Performance metrics for qualitative NTA study outputs”), we have included brief descriptions of these terms as they are used in targeted analysis, below (adapted from [38–44]).

- ***Selectivity*** describes a targeted method’s ability to detect and differentiate a unique chemical species from interferents [41, 43]. Targeted methods are developed using solutions of chemical standards with known purity, with compound-specific optimization of both the analytical method and sample preparation procedures. While nuance exists (e.g., in analytical tolerances, impact of interfering analytes), results from optimized targeted methods are generally considered unambiguously linked to a specific chemical(s). In other words, ***with targeted methods, if an analyst reports that a chemical is present in a sample, it is known to be truly present.***
- ***Sensitivity*** communicates the smallest change in concentration at which a targeted method can observe and quantify a chemical in a study sample [44]. Sensitivity is related to the limit of detection (LOD), which defines the concentration at which a chemical signal can be detected with certainty above the background [38]. Most analytical instruments have some low level of background signal or “noise” which can obscure weak/low-concentration chemical signals. ***With targeted methods, if an analyst reports a chemical at or above the LOD, it is known to be present in the sample; otherwise, it is known to be absent from the sample or present at an immeasurably low level.***
- ***Accuracy ***and ***Precision*** describe a targeted method’s ability to quantify a target chemical correctly and reproducibly. Accuracy metrics communicate the closeness of agreement between a reported concentration and a true value, whereas precision metrics communicate consistency across multiple reported values [42, 44]. Accuracy and precision are not co-dependent (any given method can be accurate, precise, or both). ***With targeted methods, if an analyst reports a chemical concentration, it is expected (at a defined level of confidence) to truly exist within a defined interval.***

For specific applications, stakeholders can select amongst targeted methods that meet clearly defined performance thresholds. Furthermore, individual labs can develop new methods or achieve certification/accreditation to conduct specific analyses with established methods, based on demonstrably acceptable performance (e.g., as established by a funding source or regulatory agency). Labs may also opt to implement existing reference methods, which are developed by federal agencies, often validated by multiple users, and carefully documented to meet specified performance requirements. Regardless of the selected approach, well-established frameworks clearly exist with which to decisively evaluate and communicate the performance of targeted analytical methods [39, 45].

## Non-targeted analysis study objectives

### Uncertainty in non-targeted analysis

**Fig. 1:**
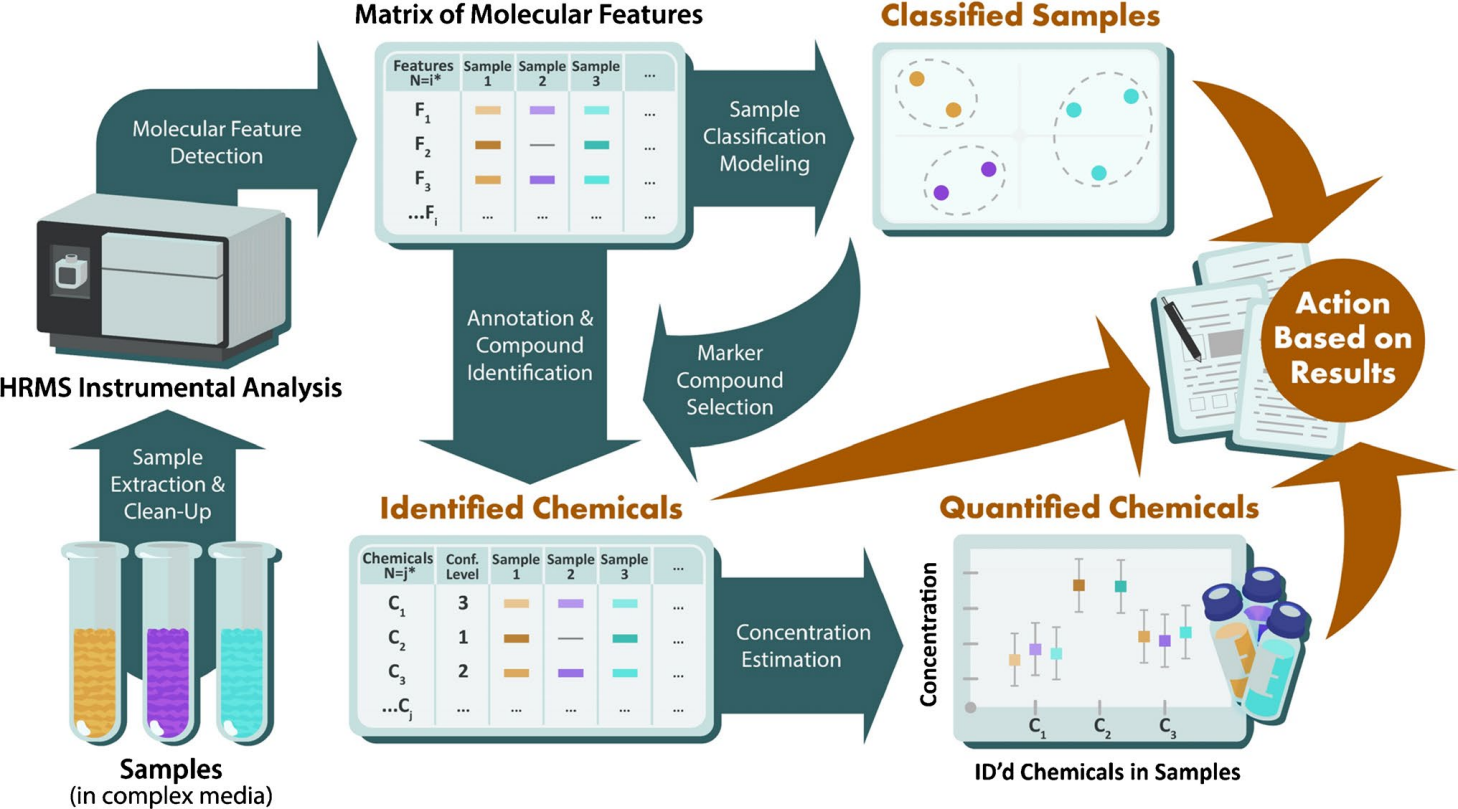
Schematic of possible NTA workflows that support three primary study objectives and corresponding study outputs discussed herein: classified samples, identified chemicals, and quantified chemicals. Note that for a given analysis, the number of features (N = i) is typically larger than the number of identified chemicals (N = j)

In contrast to targeted methods, NTA generates global chemical information for a sample. While incredibly informative, this information-rich data can be challenging to evaluate in an efficient and reliable way. NTA can support broad research objectives, with focus often on sample classification, chemical identification, and even chemical quantitation (described below and summarized in Fig. 1). Pursuit of these objectives requires experimental, data analysis, and performance assessment approaches that differ from those of targeted methods. In addition, NTA data are inherently less certain than targeted data, such that (in the absence of an available standard and/or subsequent targeted analysis):

- ***If an analyst reports that a chemical is present in a sample, it may actually be absent (e.g., it may be an isomer or an incorrect identification);***
- ***If an analyst reports that a chemical is not present in a sample, it may actually be present (e.g., it may not have been correctly identified during data processing);***
- ***If an analyst develops a sample classification model, it may not be repeatable over time and/or transferable between instruments; and***
- ***If an analyst reports a concentration, often there is no corresponding confidence interval and the true concentration could be orders of magnitude higher or lower than the reported value.***

These uncertainties prevent NTA data from being widely accepted by decision-makers. In light of this uncertainty, two urgent needs exist within the NTA research community. First, there is a need for methods that can accurately measure the extent and implications of uncertainties for any specific use case. For maximum utility, uncertainties must be characterized using standardized performance metrics and against established performance benchmarks. Second, there is a perpetual need within the community for approaches to reduce uncertainties in NTA methods. The remainder of this article focuses on the first need—establishing standardized protocols to assess NTA method performance. Specific recommendations are made for each of the aforementioned research objectives of sample classification, chemical identification, and chemical quantitation (described in detail below).

### Generating non-targeted analysis data

Many NTA studies use HRMS platforms, with each study generating MS “molecular feature” data for hundreds to thousands of (initially) unknown chemicals in study samples, with the possibility to simultaneously or subsequently collect MS/MS data (see ESM Text S2) [26, 46, 47]. Each molecular feature (often referred to as simply a “feature”) is comprised of “a set of grouped, associated mass-to-charge ratio (*m*/*z*)-retention time pairs (mz@RT) that, ideally, represent a set of MS components for an individual chemical (e.g., an individual compound and associated isotopologue, adduct, in-source product ion *m*/*z* peaks) or a single mz@RT if no such associated components are observed. A feature is represented as a tensor of observed retention time, monoisotopic mass, and intensity (e.g., peak height or peak area). Associated MS/MS product ions (ESM Text S2) may also be grouped with the MS components of a feature during HRMS data processing, depending on software algorithms” [48]. Aligning detected features across a sample set yields a data matrix of unique features × unique study samples, with sample-specific feature intensities in intersecting cells. This data matrix provides a chemical fingerprint for each sample (consisting of the individual detected features and corresponding intensities) and is the basis of all downstream NTA data analyses [26].

It is important to note that detecting more features does not necessarily indicate better chemical coverage. Larger feature numbers may indicate the true presence of more unique compounds or a failure to correctly group ions into features and/or remove artifact signals. A thorough examination of challenges associated with properly grouping ions into features and aligning features across samples (and a full discussion of corresponding feature-level performance metrics) is beyond the scope of this article; a brief topical discussion is provided in ESM Text S2.

### Sample classification

Sample classification aims to label any given study sample as belonging (or not belonging) to a specific class of interest. Rapid sample classification (particularly without time-consuming marker compound identification efforts) is of immense value to numerous private and public stakeholder groups. For example, NTA data has been useful in classifying geographic locations impacted by point source emitters [4], identifying populations affected by inadvertent chemical releases [49], flagging food items not meeting criteria for product certification [20, 50], and identifying former military service members exposed to chemical contaminants during active duty [7].

Typically, multivariate statistical analyses (e.g., principal component analysis, partial least squares discriminant analysis) and/or machine learning models (e.g., random forest, neural networks) are first built (or trained) on a “training set” of descriptor data (i.e., the aforementioned data matrix of molecular features, comprised of sample chemical fingerprints) with known sample classifications. These models (detailed discussion of which is provided elsewhere [19, 26]) can be used to determine the sample classification descriptors (e.g., presence of characteristic features, relative abundance of feature combinations) that are most effective for distinguishing the class(es) of interest. Models are then tested using the descriptor data from an additional “test set” of samples with known sample classifications (the stage at which performance assessment can occur), before application to classify unknown samples. Importantly, classification can occur without knowing the chemical/structural identity of any molecular features, although identification of “marker” or “indicator” chemicals within the chemical fingerprint (for subsequent analysis by targeted methods) is often a parallel goal (Fig. 1). Despite the benefits and increased use of NTA-based sample classification methods, there are not yet standardized methods for assessing and communicating their performance [19, 20].

### Chemical identification

Many NTA studies seek some level of chemical identification for molecular features that are prioritized by initial data analyses (e.g., through sample classification, statistical tests, temporal profiles) or prior knowledge regarding sample composition. Putative identifications are often determined using a combination of supporting data such as accurate mass, isotopic distribution, retention time, ion mobility, mass defect, and/or MS/MS product ion information, where a variety of highly integrated software tools and workflows (e.g., to perform molecular formula assignment, database/library screening, molecular networking) exist to support compound identification [51]. However, complementary manual identification efforts are often necessary, given limitations of existing spectral libraries and challenges with automated MS/MS scoring, as detailed elsewhere [52]. The final reported chemicals in a NTA study are qualified with respect to the level of identification confidence based on the amount and quality of supporting evidence (e.g., using the scale proposed by Schymanski et al. [53], where confidence level 1 = standard-confirmed and 4 = formula assignment only) (Fig. 1). Unknown/unexpected compound identification is one of the most common and best recognized advantages of NTA; for example, NTA has been used to identify natural and synthetic chemical nerve agents [13], contaminants associated with product-related illness [54] and aquatic toxicity [16], designer drugs used to enhance athletic performance [55], and chemicals released as part of industrial emissions [8] or during emergency response scenarios [56]. To date, there are no standardized approaches or benchmarks for assessing and communicating performance of NTA-based chemical identification methods. Moreover, there is no guidance as to whether performance should be evaluated at individual confidence levels or across collective results sets, hindering efforts to conduct interlaboratory performance assessments.

### Chemical quantitation

Finally, there is growing interest in using NTA for direct quantitation of chemical concentration. NTA is well-suited to prioritize emerging compounds for subsequent targeted quantitative analyses (with results effectively communicated using the aforementioned targeted performance metrics). However, analytical standards for many emerging contaminants (which include a vast array of transformation and degradation products) are not readily available via purchase or laboratory synthesis, limiting development of targeted methods. Accordingly, quantitative NTA (qNTA) provides an opportunity to provisionally estimate chemical concentrations both in prepared sample extracts (i.e., translating instrument response to concentration in the absence of chemical standards) and in the original sampled media (i.e., accounting for losses during sample processing without structurally paired, isotope-labeled recovery standards) (Fig. 1). To achieve this end, most qNTA methods either apply calibration data from structurally similar compounds to newly discovered analytes, or use response modeling approaches to estimate the ionization behaviors of observed compounds given predicted structures and related physicochemical properties [37, 57]. Although few qNTA use cases exist, notable examples include using qNTA to estimate chemical weight fractions in consumer products [27], concentrations of emerging PFAS in soil/surface water [4], concentrations of pesticides and mycotoxins in cereals [58], concentrations of micropollutants in groundwater [59], and concentrations of natural products [60]. However, limited methods to estimate the uncertainty of any given qNTA prediction, and a lack of recommended performance metrics, currently hamper broader implementation of qNTA.

## Non-targeted analysis performance assessment

Given the limited guidance for evaluating and communicating overall NTA method performance for the aforementioned NTA study objectives, the following sections propose initial strategies and discuss current limitations of these approaches. Two of the NTA study objectives described above (sample classification and chemical identification) correspond to qualitative study outputs, while the third is quantitative. The discussion below is separated accordingly, as metrics for qualitative and quantitative outputs are necessarily distinct.

Additionally, regardless of the NTA study objective, two key distinctions between NTA and targeted analysis performance assessments should be noted. First, method optimization and performance assessments for targeted methods are specific to the targeted chemical(s) of interest (i.e., the same chemical(s) to which the method will be subsequently applied in unknown samples). In other words, targeted methods have a clearly defined domain of applicability. In contrast, NTA methods are necessarily less specific, because the chemicals of interest in an unknown sample set are not decided in advance of sample preparation and data acquisition. Thus, thorough quality assurance and quality control (QA/QC) procedures remain critical in NTA (and are discussed elsewhere [30–33]), and the domain of applicability for a given NTA performance assessment (discussed further below) should be considered.

Second, real samples analyzed in NTA studies are often chemically complex, with unknown (or partially known) composition. Accordingly, performance evaluations for all NTA methods will likely rely on suites of test samples for which the true values are known (i.e., with established sample classifications, known chemical identities, and/or quantitative concentrations). Unlike the reference materials available for assessment of many targeted analytical methods, standardized test samples for NTA performance assessments are not yet available and will be challenging to develop (e.g., determining the needed complexity, chemical coverage, etc. to support effective performance assessments and comparisons). However, as NTA data becomes more widely used and accepted, development of such test samples may enable performance validation across laboratories and over time.

## Performance metrics for qualitative NTA study outputs

**Fig. 2:**
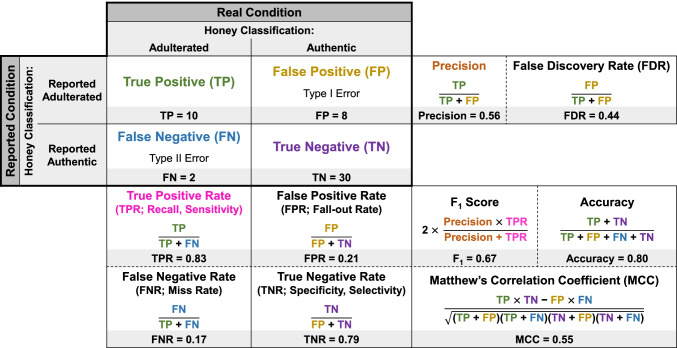
The confusion matrix and associated performance metrics. The core confusion matrix is within the bold outline; additional boxes provide formulas for calculating associated performance metrics. Dashed lines between metrics indicate interchangeable or tandem usage. Text color on certain classifications/metrics facilitates tracking use of those values in calculations and is not intended to highlight certain metrics as more important. A sample classification example is provided in the shaded gray cells for a test set of 50 honey samples (including 12 that are adulterated with non-honey sugar sources and 38 that are considered authentic, > 99.99% honey), where the classification model correctly reports 10 as adulterated and 30 as authentic but incorrectly reports 8 as adulterated and 2 as authentic

For NTA-based sample classification and chemical identification performance assessments, we focus on use of the confusion matrix and associated metrics. The confusion matrix is an error matrix often presented as a table of true positives (TPs), true negatives (TNs), false positives (FPs), and false negatives (FNs) (bold outline, Fig. 2). Calculation of associated metrics based on the quantity of true/false positives and negatives serves to summarize the results and describe the method accuracy, precision, sensitivity, and selectivity (Fig. 2) [29]. Importantly, we note that these terms are identical to those used to describe targeted analytical method performance (described earlier). However, in the context of the confusion matrix, the same terms are used to define various calculated metrics. This terminology is well-established; the confusion matrix has been routinely applied in other fields to assess performance of analytical or biological tests with discrete, often binary, qualitative outputs (e.g., tests to detect whether a person does/does not have a disease) [61–64]. Therefore, awareness of distinct uses of these terms for targeted and non-targeted applications is necessary. Here, we distinguish meaning by capitalizing the terms when describing calculations from the confusion matrix (e.g., Accuracy, Precision). We first present the confusion matrix in the context of sample classification, then examine its applicability to evaluating chemical identification performance.

### Sample classification performance

For sample classification applications, performance is based on the ability of a method to correctly classify or group samples into a binary (or higher order) system (e.g., adulterated vs. authentic food, agricultural vs. urban runoff, wines of various origins) [65–67]. Importantly, NTA-based sample classification efforts are directly analogous to established sample classification approaches that use data from other analytical methods (e.g., targeted analytical data, nuclear magnetic resonance (NMR) data, image analyses) [68, 69]. Thus, given advanced knowledge of the correct classification (known test samples) and sufficient statistical power (driven by the number of samples), application of a confusion matrix to assess performance is relatively straightforward and follows established practices [19, 70].

Use of the confusion matrix requires clear definition of the positive and negative conditions. These positive and negative conditions are often not balanced, and convention dictates that the rarer or more interesting class (e.g., the adulterated state, in a study classifying adulterated and authentic food) is designated the positive condition. True positives (TPs) are correctly reported as the positive condition (an adulterated sample correctly classified as adulterated), while true negatives (TNs) are correctly reported as the negative condition (an authentic sample correctly classified as authentic). Likewise, false positives (FPs) are incorrectly reported as the positive condition (an authentic sample incorrectly classified as adulterated, representing Type I errors) and false negatives (FNs) are incorrectly reported as the negative condition (an adulterated sample incorrectly classified as authentic, representing Type II errors). The total number of true/false positives and negatives must sum to a known, discrete number that bounds the confusion matrix. For sample classification, the performance assessment boundary is the total number of samples in the test set. The suite of associated performance metrics includes the True Positive Rate (TPR; also termed Sensitivity or Recall), False Negative Rate (FNR; Miss Rate), True Negative Rate (TNR; also termed Specificity or Selectivity), False Positive Rate (FPR; Fall-out Rate), Precision, False Discovery Rate (FDR), Accuracy, F_1_ Score, and Matthew’s Correlation Coefficient (MCC) (calculations detailed in Fig. 2). We note that this set of metrics/terminology includes those most relevant for NTA and is not exhaustive; detailed resources exist elsewhere [71, 72].

The individual metrics are often grouped into pairs or sets that are used interchangeably or in tandem (separated by dashed lines in Fig. 2; for additional detail, see ESM Text S3) [73]. For example, Accuracy, F_1_ Score, and MCC are often used in combination to provide a well-rounded description of performance, particularly in the case of unbalanced datasets (i.e., those with many more observations in one class relative to the other(s)) [74, 75]. Consider the classification of a test set of 50 honey samples (including 12 that are adulterated with non-honey sugar sources and 38 that are considered authentic, > 99.99% honey): for a classification model that correctly reports 10 as adulterated and 30 as authentic but incorrectly reports 8 as adulterated and 2 as authentic, the confusion matrix has 10 TPs, 30 TNs, 8 FPs, and 2 FNs (Fig. 2). However, the dataset is unbalanced (i.e., more samples are authentic [*n* = 38] than adulterated [*n* = 12]). Thus, although calculated Accuracy (0.80) indicates acceptable model performance, the F_1_ Score (0.67; where 1 indicates perfect Recall and Precision, see ESM Text S3) and MCC (0.55; where − 1 indicates perfect misclassification, 1 indicates perfect classification, and values around 0 indicate random guesses, see ESM Text S3) are much lower, emphasizing that considering Accuracy alone could produce greater confidence in a model than is warranted.

Overall, while it is relatively straightforward to apply the confusion matrix to describe sample classification model performance, many challenges are associated with developing robust, reproducible NTA-based classification models using HRMS data. First, it is often difficult to obtain training/test sets of truly known/certified samples that both contain sufficient sample quantities [76] and are well-matched to the potential variability in unknown samples (e.g., different brands of food, different regions for environmental samples) to appropriately build and test models [20]. Similarly, it is important to precisely define the classes of interest based on the goals of the study and to ensure that the samples are truly representative. In the honey authentication example, there are a variety of ways a honey could be adulterated (e.g., different types of non-honey sugar sources) and at varying levels (e.g., 80% honey vs. 50% honey). Second, calculated performance metrics are not reliable when a model is implemented outside its domain of applicability (e.g., applying a model built and tested only on Gala apples to Red Delicious apples) [20]. Third, the number of model variables (i.e., detected features, often 1,000’s—particularly for chromatographically separated HRMS data) is typically far greater than the number of samples (usually 10’s to 100’s), which can lead to data overfitting and overinterpretation [19, 20, 76]. Finally, classification models are sensitive to small differences in the data that are not specific to the variable of interest, such as changes in retention time and background ions associated with different instrumental set ups (or even use of the same instrument over time, given changes in solvents, analytical columns, or other analytical conditions). Therefore, applying models long-term and transferring models between instruments/labs can be challenging [19]. Performance validation for NTA-based sample classification may necessarily remain specific to a given laboratory (or instrument) until robust methods for isolation of reproducible feature lists are further advanced (see ESM Text S2).

### Chemical identification performance

For chemical identification, the boundary of the performance assessment is the known, discrete number of considered chemicals. The positive and negative conditions in the confusion matrix correspond to compounds known to be present in or absent from a sample, respectively, within that defined boundary. Importantly, establishing the confusion matrix boundary for chemical identification performance assessments is significantly more challenging than for sample classification, as more options exist, and the size of the boundary (i.e., the number of considered chemicals) can substantially impact the calculated metrics. For example, theoretical boundaries for chemical space within a study might include all spiked compounds in a sample, all compounds detectable by a given data acquisition method, all compounds in a suspect screening database of 100, 500, or 10,000 compounds, or even all possible chemicals. However, certain boundaries are not well understood, such as those in which the total number of compounds considered is not readily defined (e.g., all compounds detectable by a certain data acquisition method or all possible chemicals). Use of a confusion matrix in these cases is not practical.

Additionally, the domain of applicability—the chemical space to which the performance assessment results are relevant—should also be considered and is closely related to, but distinct from, the performance assessment boundary. For example, for a performance assessment bounded by a spiked mixture of 100 PFAS compounds, the domain of applicability could be defined as PFAS compounds that are structurally similar to those in the test sample (i.e., performance results do not necessarily indicate expected performance for other compound classes, such as pesticides). Note, the availability and affordability of analytical standards will determine the breadth of performance assessments relying on known chemical mixtures.

Importantly, setting universal performance criteria within a constrained chemical space could bias NTA methods and create analytical blind spots that unintentionally miss toxicologically relevant but as-yet unknown chemicals, thus degrading the long-term utility of NTA studies. However, given the diversity of sample types, contaminants of interest, and study goals, selecting a universally accepted test mixture (or pre-determined chemical space) to bound NTA performance assessments remains challenging. Even when more complex samples are used (e.g., representing broader chemical space, a range of ionization efficiencies, different matrix backgrounds), there are not yet established tools and metrics by which to translate performance in known mixtures to that in real samples (nor to determine the necessary complexity to adequately represent performance in real samples).

**Fig. 3:**
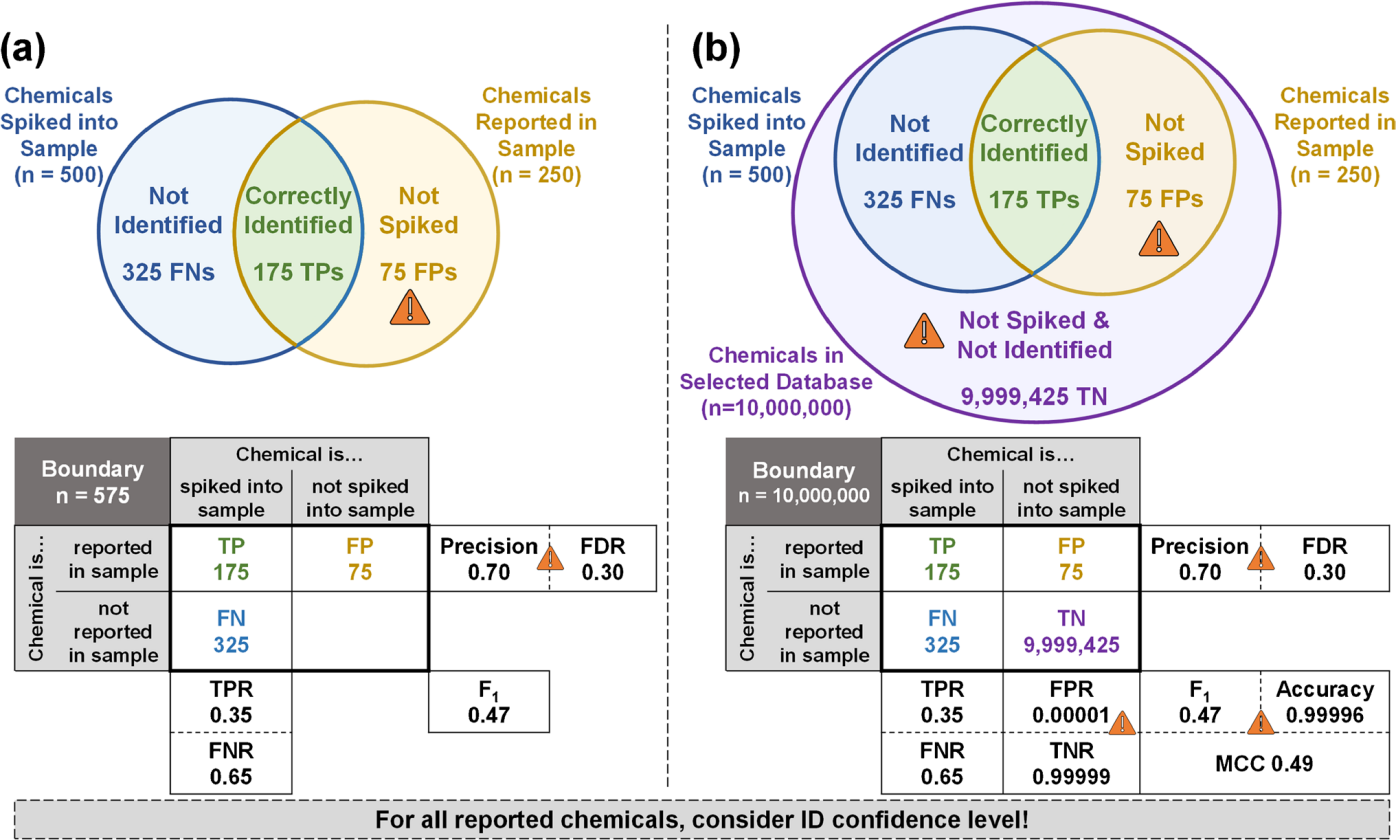
Venn diagram and confusion matrix representations of chemical identification performance assessment approaches, with a numeric example provided. Orange caution symbols indicate values where caveats should be carefully considered. (**a**) The approach considers only the chemicals spiked into the sample (blue circle) and reported in the sample (yellow circle), allowing calculation of metrics relying solely on TP, FP, and FN. (**b**) The approach from (**a**) is modified to include a database of interest (purple circle), allowing determination of TN and calculation of all performance metrics. Additional significant figures are listed for some metrics (i.e., FPR, TNR, Accuracy) to clearly provide exact values, given the number of TNs used in the calculation

Thus, although performance assessment requires samples with known compositions (e.g., generated standard mixtures or well characterized mixtures), we do not attempt to define the type of test samples or chemical space that should be used for chemical identification performance assessments. Instead, the discussion below focuses on defining two recommended, feasible boundaries for use in confusion matrix–based performance assessments: (1) the total number of chemicals known to be present and/or reported by the analyst in a sample set, and (2) the total number of compounds in a suspect screening database. Additionally, we present important considerations for applying those boundaries to the test sample(s)/chemical space of choice, with recommendations for current best practices. To support the discussion, Fig. 3 depicts chemical identification performance assessment examples using these two boundaries for a test sample with 500 spiked compounds.

#### Boundary 1: Compounds known to be present and/or reported

The first recommended performance assessment boundary considers only the unique chemicals known and/or reported to be present in the test sample (*n* = 575 in Fig. 3a), where only one chemical is reported per feature (see section titled “Considering identification confidence level”). The list of chemicals known to be present in the sample (often a list of spiked compounds) defines the combined number of TPs and FNs in the confusion matrix (e.g., *n* = 500 in Fig. 3a), while the list of reported chemicals defines the combined number of TPs and FPs (e.g., *n* = 250 in Fig. 3a). To apply this approach to performance assessment, the lists of chemicals known to be present and those reported in the sample must be cross-referenced using the available evidence, such that, at most, one reported chemical is assigned to each chemical known to be present in the sample (additional nuance regarding “chemicals known to be present” is further discussed in the section titled “Additional considerations for assigning TPs, FPs, and FNs” below). Then, a spiked chemical that is reported as present is denoted a TP, a spiked chemical that is not reported is a FN, and a reported chemical that was not spiked is a FP (in Fig. 3a: 175 TP, 325 FN, and 75 FP). With this boundary, TNs cannot be determined because a set of chemicals that are *not* present/reported in the sample is not defined. Thus, only performance metrics that use TP, FN, and FP can be calculated (i.e., TPR/FNR and Precision/FDR). Performance metrics that rely on TNs (e.g., TNR/FPR and Accuracy) cannot be calculated. For method evaluation, TPR and Precision indicate the proportion of chemicals spiked into the sample and the proportion of chemicals reported in the sample that are TPs, respectively. A composite performance metric, F_1_ Score, can also be calculated and may be useful for comparing method performance in cases where TPR and Precision have equal importance, although this will not always be the case in NTA studies.

In general, focusing the assessment on TPR/FNR and Precision/FDR supports a balanced evaluation of method performance. TPR and FNR rely only on the quantity of TPs and FNs (bounded by the number of known chemicals in the sample), allowing interpretation in the context of a practical, understandable quantity [77]. However, in considering these metrics alone, there is no penalty for over-reporting (i.e., “shooting in the dark” to report as many identifications as possible, with the hope of achieving more TPs), because FPs are not used in the TPR/FNR calculations. For example, consider the case where Lab I reports 250 total chemicals (i.e., Fig. 3) and Lab II reports 1000 total chemicals. If both labs report 175 TPs and 325 FNs, both achieve TPR = 0.35 and FNR = 0.65, even though a much smaller proportion of total chemicals reported by Lab II were TPs (II = 175/1000 vs. I = 175/250). Thus, although use of TPR/FNR alone may be deceiving, the complementary evaluation of Precision/FDR indicates the extent of over-reporting because FPs are considered in these calculations. Continuing the example above, Lab I’s Precision = 0.70 and Lab II’s Precision = 0.18, effectively illustrating the over-reporting behavior of Lab II. The application and goals of the study may dictate the most important metrics for comparing method performance. For example, emphasis on Precision over TPR may be warranted if over-reporting (FPs) is of higher concern than missed/incorrectly identified chemicals (FNs) and vice versa. If minimizing both FPs and FNs are of equal importance for the study, then the previously mentioned F_1_ Score can also be used. In any case, it is advisable to determine TPR/FNR and Precision/FDR for a balanced assessment of method performance.

#### Boundary 2: A suspect screening database

The second recommended performance assessment boundary uses a suspect screening database (e.g., *n* = 10,000,000 in Fig. 3b). For this approach, the chemicals known to be present and/or reported in the test sample must be included within the selected database (as is assumed in the example here). We note that the database content may vary (e.g., containing only molecular formulas vs. MS/MS spectra); we discuss considerations with respect to identification confidence below (see “Considering identification confidence level”). TPs, FNs, and FPs are assigned as described above for the known and/or reported boundary (e.g., 175 TP, 325 FN, and 75 FP in Fig. 3b). Additionally, chemicals in the suspect screening database that are neither known to be present nor reported are TNs (e.g., 9,999,425 TN in Fig. 3b). Thus, this boundary allows completion of the full confusion matrix and calculation of all associated performance metrics. This approach was implemented by Nuñez et al. (2019), where they used all chemicals present in the EPA ToxCast library (*n* = 4,737) to bound their identification performance assessment during EPA’s Non-Targeted Analysis Collaborative Trial (ENTACT) [78], as each synthetic test sample was known to contain a subset of the ToxCast chemicals [52].

Importantly, the quantity of TNs is highly dependent on the size of the selected database. The selection of a very large database can bias performance metrics that are calculated using TN (e.g., yielding artificially high TNR and Accuracy, and artificially low FPR) [70]. For example, the large database used in the example depicted in Fig. 3b yields TNR = 0.99999, FPR = 0.00001, and Accuracy = 0.99996; in contrast, the TPR is only 0.35. As noted above for sample classification with unbalanced datasets, use of F_1_ Score (0.47) and MCC (0.49) alongside Accuracy provides a better indication of method performance than Accuracy alone.

Additionally, some databases may include compounds that are not detectable by a given method (e.g., due to sample preparation choices or analytical method settings), limiting the utility of classifying such compounds as TNs. For example, an LC–MS method should not get credit for “TNs” that are not LC-amenable (e.g., many volatile GC-amenable chemicals), even if they are truly not present in the sample. On the other hand, intentional use of a specific database or library thought to be more relevant to the study (e.g., that covers a select subset of known chemical space) to bound a performance assessment may allow for a more realistic estimate of TNs (by excluding irrelevant compounds from consideration), but may also limit the domain of applicability of the assessment (e.g., other compounds that may have been detected are not considered) [52]. Overall, although this approach to performance assessment enables completion of the confusion matrix, we still recommend caution when interpreting the total number of TNs and TN-derived metrics.

#### Additional considerations for assigning TPs, FPs, and FNs

Depending on the composition of the spiked mixture or test sample used for performance assessment, additional nuance may be required regarding TP, FN, and FP assignment (regardless of the performance assessment boundary). In complex samples (even those made with neat, spiked standards), it is difficult to definitively know every compound that is or is not present (due to trace impurities, in-vial transformation of unstable compounds, etc.) [77, 79]. *Thus, for the purposes of a chemical identification performance assessment, the list of compounds defined as truly present in a sample is determined by the extent to which the sample is initially characterized (e.g., if the sample is analyzed, verified impurities, isomers, transformation products, *etc*. may be included in the list of compounds defined as truly present).* This approach eliminates ambiguity in assigning TPs and FNs, as each TP and FN must correspond to an individual, known chemical in the sample.

Here, we define the subset of reported compounds that are truly present in the test sample (e.g., as degradants, reaction products, and/or impurities), but are not known to be present, as “unintentional TPs” (uTPs). Differentiating uTPs from FPs and confirming true presence/absence in the test sample is often not practical, given necessary analyst time and resources and the limitations of available databases and spectral libraries. In the relatively simple example of a neat standard mixture in Fig. 3, there are 75 FPs, some of which may be uTPs. However, using samples with greater chemical complexity for performance assessments could yield 100’s of uTPs. The distinction between FPs and uTPs may be particularly challenging in test samples with large quantities of constitutional isomers and/or stereoisomers, as a laboratory may assign the same identification to multiple features. In such cases, we again recommend using the stated composition of the test sample to assign TP/FP/FN. For example, if the presence of a constitutional isomer is known (e.g., it is known that dibutyl phthalate is present, but diisobutyl phthalate is not), a laboratory must report that same constitutional isomer (e.g., dibutyl phthalate, not diisobutyl phthalate) to achieve a TP. Likewise, if multiple stereoisomers are not specified for a known compound in the test sample (e.g., the alkaloid piperine, which exists as four stereoisomers), but a laboratory reports multiple identifications of that compound—only one TP is counted, and the remainder are considered FPs (e.g., if four piperine identifications are reported, it is considered 1 TP and 3 FPs). *Treating uTPs as FPs when calculating Precision and FDR is practical and ensures that conservative values for these performance metrics are reported.* To balance this conservative approach, a laboratory could present evidence to indicate that a given percentage of reported FPs are in fact uTPs.

Finally, we note that a laboratory could perform additional follow-up evaluations to examine the up-stream factors driving observed overall performance. For example, a FN might occur if the chemical was not detected by the selected instrumental method or if a detected feature was not assigned the correct identity by the data analysis workflow. Likewise, a FP might occur if the molecular formula was annotated correctly, but the incorrect chemical name was assigned (e.g., if the correct chemical was not included in the selected suspect screening database or ranked lower based on available metadata/literature citations, or if the MS/MS spectra was interpreted incorrectly). *Ultimately, if such evaluations result in changes to the laboratory’s method, performance should be reassessed by the same approach and metrics described above*.

#### Considering identification confidence level

The performance metrics associated with the confusion matrix give equal weight to each reported compound. In most NTA studies, however, chemicals are reported at different levels of identification confidence (such as those described by Schymanski et al. [53]). This presents a dilemma for how best to implement the confusion matrix given different levels of uncertainty in chemical identity assignments. Consider two hypothetical laboratories that produce the same performance metrics depicted in Fig. 3 (e.g., TPR = 0.35, Precision = 0.70). Hypothetical lab #1 indicates that 100% of reported TPs have a confidence level of 1 or 2. Alternatively, hypothetical lab #2 indicates that 50% have a confidence level of 1 or 2, and 50% have a confidence level of 3. In the absence of consideration for these reported confidence levels, the two labs would have equal performance. Yet, with additional interpretation of confidence levels, a stakeholder could readily differentiate the capabilities of the two labs. All reported compounds (i.e., TPs and FPs) should be assigned a confidence level, which then enables calculation of the corresponding Precision and FDR metrics separately for each confidence level. As a theoretical example, confidence level–specific metrics could allow a lab to demonstrate Precision of 0.95 for level 2 identifications, despite an overall Precision of 0.70. This approach was employed by Nuñez et al. in their ENTACT performance evaluation to demonstrate that FDR was inversely related to the amount of available evidence supporting each identification [52]. As such, *identification confidence should be incorporated into performance assessments by enumerating the proportion of reported compounds (i.e., TPs and FPs) and the corresponding Precision/FDR metrics for each confidence level.* This approach does not impact performance metric calculations (Fig. 3), but instead provides additional nuance to their interpretation. We note that it is conceptually challenging to generate separate confusion matrices for each confidence level because chemicals that are not reported present (FNs and TNs) cannot be assigned confidence levels. However, we anticipate that future iterations of performance assessment approaches may still seek to separate confidence levels out of practical need (for example, separately considering performance for identifications associated with a chemical structure [levels 1–3] versus those associated with only a molecular formula [level 4]).

As noted above, confusion matrix–based performance calculations require that only one reported chemical corresponds to each chemical known to be present in the sample. We note that when an identification confidence below level 1 is achieved for a given feature, laboratories may provide multiple possible chemical identities that correspond to that single feature (e.g., a list of chemical identifiers associated with a level 4 molecular formula assignment; two potential isomers associated with a level 3 identification). Such an approach may be informative to a stakeholder (e.g., a risk-assessment scenario in which available resources are limited and subsequent chemical identification efforts must be prioritized). However, in the case of performance assessments, the result for each feature must be reduced to a single reported identification (e.g., a single isomer with level 3 confidence) to enable use of the confusion matrix. Further debate in the community is needed to determine best practices for chemical identification performance assessment with respect to reporting a single result versus a list of possible chemical identities for a given observed feature. Further development of guidance to address challenges associated with incorporating identification confidence during performance assessment will be critical to ensure valid assessments and comparisons of method performance.

## Performance metrics for quantitative NTA

### Understanding targeted and non-targeted approaches to quantitation

**Fig. 4:**
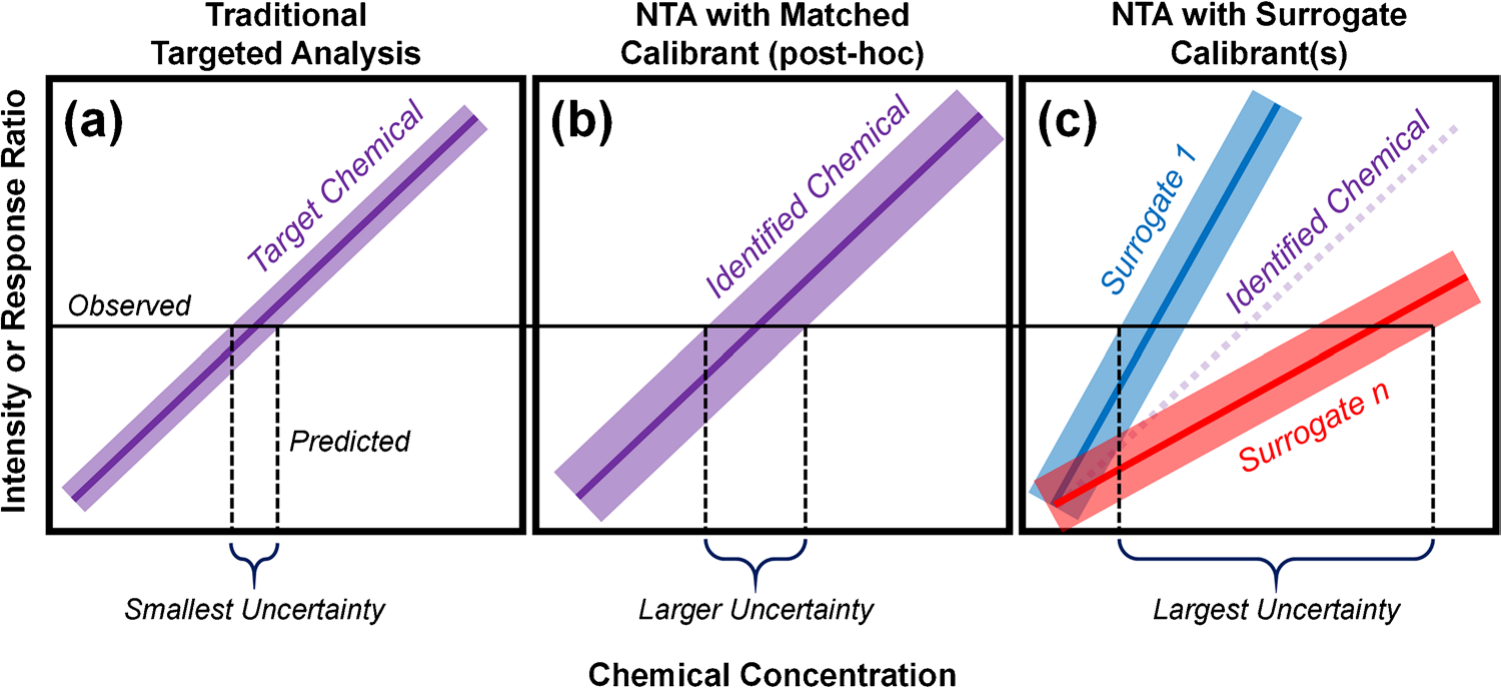
Comparison of the degree of uncertainty associated with concentration estimates obtained from: (**a**) a targeted analytical method that was developed and optimized for the target chemical (smallest uncertainty), (**b**) a NTA method that putatively identified the chemical, after which a standard was obtained, the chemical was confirmed, and post hoc quantitation was performed, and (**c**) a NTA method that putatively identified the chemical, after which surrogate chemical(s) (that were not structure-matched to the putatively identified chemical) were used for quantitation (largest uncertainty). The solid lines (and dotted line in (**c**)) correspond to regression lines and the shaded area about each line corresponds to prediction intervals for the respective regression line. Although 2 surrogate chemical calibration curves are depicted in (**c**), any number (*n*) of surrogate chemicals may be used to estimate concentration. The purple dotted line in (**c**) indicates the theoretical calibration curve of the putatively identified chemical

All quantitative estimates from both targeted and non-targeted mass spectrometry analyses contain uncertainty. In targeted studies, established approaches exist to minimize this uncertainty (and maximize the accuracy and precision of quantitative estimates), including use of calibration curves, structurally paired internal (recovery) standards, and batch corrections. A calibration curve—a mathematical relationship between known concentration and measured intensity of a given analyte [40]—enables direct calculation of target analyte concentration in any new sample (Fig. 4a). Clear guidance exists regarding the development and use of calibration curves for target analyte quantitation [39, 45]. This guidance relates to selection of appropriate concentration ranges; the number, spacing, and replicates of calibration curve points; the utilization of paired internal standards; and mathematical procedures (e.g., weighting, transformation) used to meet calibration model assumptions (e.g., linearity, homoscedasticity). All guidance is meant to ensure that quantitative predictions are accurate and can be defensibly bounded within a calculable error range (e.g., a 95% confidence interval).

Most NTA studies consider quantitative estimation procedures only after confirming the presence of specific analytes. With this post hoc approach, many decisions made by the analyst will affect the certainty of eventual quantitative estimates. For example, an analyst may wish to use the existing intensity measures of an analyte (produced from the original NTA experiment) as the basis for quantitation vs. rerunning study samples alongside a calibration standard. Likewise, if rerunning study samples and reacquiring intensity measures, an analyst may opt to preserve the initial NTA instrument method vs. developing an optimized method for the recently discovered analyte(s) (more akin to a targeted analysis). Finally, to control for sample recovery effects and/or batch effects, an analyst may opt to interpret analyte intensity measures after adjustment for surrogate intensities (as a response ratio). Surrogates are any measured compounds that are used to calibrate the concentration of the detected analyte; they may be an isotopically labeled version of the analyte or a different compound (native or labeled). In the latter case, the structural similarity between the selected surrogate and the targeted analyte will influence the error in the eventual quantitative predictions [60].

Both targeted and post hoc quantitative methods involve the use of chemical standards to quantify high-interest analytes. As such, traditional practices involving chemical-specific calibration curves can be implemented. However, compared to targeted methods, greater uncertainty is expected for post hoc quantitation because more sources of variability often remain uncontrolled (e.g., recovery, matrix effects, peak integration) (Fig. 4b). For the purposes of performance evaluation, it is critical that all sources of variability are considered when reporting quantitative estimates to stakeholders. As is customary for targeted analysis, confidence intervals (or other measures of numerical uncertainty) should accompany each quantitative estimate for each identified chemical to clearly communicate the range in which a chemical concentration is truly expected to lie. For example, using a targeted method, a chemical may be reported to have an estimated concentration of 10 ng/mL with a 95% confidence interval of 8 ng/mL (lower bound) and 12 ng/mL (upper bound). Using a post hoc quantitative method, the same chemical in the same sample may again have an estimated concentration of 10 ng/mL, but with a 95% confidence interval of 1 ng/mL (lower bound) and 20 ng/mL (upper bound). By clearly communicating the uncertainty about the prediction, analysts and stakeholders can make an informed decision as to what action(s) may be needed.

In contrast to quantitation with targeted or post hoc approaches, we define quantitative NTA (qNTA) methods as those that generate concentration estimates in the absence of reference standards for the compounds of interest. Numerous qNTA methods have been developed to support chemical surveillance applications, with several recent articles providing excellent summaries of the various qNTA (or “semi-quantitative”) strategies [37, 80–83]. The most basic and commonly used approach for qNTA involves surrogate calibration, where calibration information for one or more surrogate analytes are established using the selected analytical method and are then applied to newly identified chemicals to generate concentration estimates (Fig. 4c). Numerous approaches exist for surrogate selection, ranging from use of a single surrogate analyte for all qNTA predictions [27], to optimized surrogate selection for each newly detected analyte [60]. Model-based qNTA methods also exist in which structural descriptors (e.g., molecular surface area) and physiochemical properties (e.g., log*P*, p*K*_a_) are used to predict the ionization efficiency (IE; the number of gas-phase ions generated per mol of the compound) of each identified analyte [82]. Yet, even with model-based methods, empirical measures of surrogate chemicals are still needed to calibrate IE estimates to the selected analytical platform/method [59, 80, 82]. As such, all qNTA methods, in some capacity, draw on existing experimental data for selected surrogate analytes.

Calibration information about each surrogate analyte is often distilled into a single numeric value, termed a “response factor” (RF) [80, 82]. Mathematically identical to a calibration curve slope for targeted analyses (under conditions of no experimental error), each chemical-specific RF is the quotient of an observed instrument intensity and the corresponding known concentration. The RF for a given surrogate calibrant (which may be normalized using an internal standard) is often assumed stable within the linear dynamic range (i.e., the range in which chemical concentration is directly proportional to instrument response [44]), allowing concentration predictions for each newly identified analyte (where estimated concentration = observed intensity / surrogate calibrant RF). In reality, RF is never a perfectly stable parameter, with measurements fluctuating both between and within chemicals, and is often impacted by analytical matrix effects. Given these fluctuations, the resulting qNTA estimates are subject to the sources of error described above for targeted analyses (within chemical), as well as additional sources of variance (between chemical). Overall, the magnitude of error is expected to be largely driven by the chemical similarity (or dissimilarity) between the selected surrogate(s) and the analyte for which concentration estimates are being generated, with the potential for additional error driven by analytical matrix effects that may differentially impact individual chemicals (Fig. 4c).

### Quantitative performance assessment approaches for NTA

As with qualitative NTA methods, the performance of any qNTA method must be evaluated using chemical test sets. With the simplest evaluations, each chemical in the test set has a known concentration and a concentration estimated by a qNTA method. The error associated with each chemical is calculated as the quotient of the estimated and known chemical concentration. Given a vector of error estimates across the test set, it is common to report the central tendency (e.g., mean absolute error) and maximum observed error [59, 80]. It is also common to report measures of statistical agreement between predicted and known values (e.g., *R*^2^ or *Q*^2^) [60, 84]. These performance metrics communicate critical information about the suitability of a qNTA method for a given application. Yet, they do not communicate confidence in any new concentration estimates for any newly detected analytes. *As such, researchers are encouraged to develop methods to communicate the uncertainty in all qNTA concentration estimates, as is common practice for traditional targeted methods.*

Groff et al. recently explored two qNTA approaches to estimate uncertainty in concentration predictions for newly detected analytes, which is fully explained in [85], thus a brief description is provided in this article for context. Their initial “naïve” approach estimated the 2.5^th^ and 97.5^th^ percentiles of a global RF distribution (i.e., a distribution of RF values collected across a large chemical training set, with multiple RF values per chemical) [85]. These outer percentile estimates were applied to intensity measures from chemical test sets to yield concentration estimates with 95% confidence intervals. Using five-fold cross validation, ~ 95% of true concentration values were shown to be contained within the estimated confidence intervals (which spanned two orders of magnitude). Confidence intervals were ultimately narrowed using a second qNTA method, which used chemical IE estimates to restrict the range of likely RF values for each analyte. To our knowledge, the work of Groff et al. is the first and only to estimate concentration confidence limits for individual chemicals using NTA data collected via semi-automated methods.

While the methods of Groff et al. serve as a useful proof-of-concept to guide future qNTA endeavors, the reported magnitude of prediction error does not reflect a “real-world” analytical scenario, since all considered chemicals were prepared in organic solvent. True NTA studies will experience additional variability stemming from sample recovery and matrix effects. Further experimentation is therefore needed to quantitatively examine these well-known sources of error, and to ultimately develop qNTA best practices that minimize error. While there have been efforts to correct for matrix effects causing ion suppression during HRMS detection by electrospray ionization [86–89], to the best of our knowledge, no models exist to predict matrix effects and recovery efficiencies based on analyte structure and experimental conditions during sample preparation. Given these information gaps, real-world measurement error is likely to remain largely unconstrained in qNTA studies for the foreseeable future. Regardless of the magnitude of error, qNTA applications must strive to bound all quantitative predictions to best enable confident stakeholder decisions; we emphasize the urgency of developing effective approaches in support of this goal.

## Conclusions: The future of NTA performance assessment

Non-targeted analysis using HRMS can provide a vast amount of information regarding sample chemical composition. Given the diversity of approaches that can be used to mine information-rich NTA data, QA/QC, performance assessments, and awareness of possible sources of bias in assessing overall NTA method performance are critical to promoting accurate reporting and supporting critical evaluations by researchers, colleagues, reviewers, and editors. Improved communication regarding the performance of NTA methods will increase implementation of NTA methods and enable effective comparisons of NTA results and the use of NTA data for meaningful decision-making. We believe the discussion presented here sheds light on the topic of performance in NTA studies, both demonstrating the need to implement performance assessments in NTA and providing an initial framework for further discussion in the community.

Of course, additional research efforts are needed to address the knowledge gaps discussed above (e.g., approaches to enable and evaluate interlaboratory transferability of sample classification models, guidance regarding chemical identification performance assessments across identification confidence levels, the necessary complexity of test samples, approaches/tools to assess and describe observable/identifiable chemical space, methods to bound NTA-derived quantitative predictions in real samples). Ultimately, there may even be a need to develop more suitable performance assessment metrics that are potentially distinct from those presented here. We believe the development and availability of broadly accepted chemical standards (or criteria that laboratories could use to develop their own in-house standards) for use in NTA performance assessments is a critical step. These things are necessary in order to consider developing accreditation standards for NTA like those that exist for targeted analyses. We hope the NTA community continues to debate and converge upon best practices for evaluating NTA method performance that will reduce confusion, support accurate communication, and enhance broad usability of shared data for key stakeholders.

## Supplementary Information

Below is the link to the electronic supplementary material.

Supplementary file1 (DOCX 54 KB)

## Declarations

### Disclaimer

Views expressed in this article are those of the authors and do not necessarily represent the views or policies of the US Food and Drug Administration (FDA), the US National Institute of Standards and Technology (NIST), the US Environmental Protection Agency (EPA), or the Southwest Research Institute (SwRI). Commercial equipment, instruments, or materials may be identified in this research to adequately specify experimental procedures. Such identification does not imply recommendation or endorsement by the aforementioned institutions, nor imply that the materials or equipment identified are necessarily the best available for the purpose. FDA, NIST, EPA, and SwRI collaborated in the research described here. It has been subjected to internal agency reviews and approved for publication.
