# Supplementary material for: Approaches for assessing performance of high-resolution mass spectrometry–based non-targeted analysis methods
Source: Anal Bioanal Chem. 2022 Jul 7;414(22):6455–71. doi: 10.1007/s00216-022-04203-3 (PMC9411239; doi:10.1007/s00216-022-04203-3)
Supplement: Supplementary file 1 — Supplementary file1 (DOCX 54 KB) [file 216_2022_4203_MOESM1_ESM.docx]

**Supporting Information**

***Analytical and Bioanalytical Chemistry***

**Approaches for Assessing Performance of High-Resolution Mass Spectrometry-based Non-Targeted Analysis Methods**

Christine M. Fisher^1^* (0000-0001-5612-5281)_,_ Katherine T. Peter^2,3^* (0000-0001-7379-265X), Seth R. Newton^4^ (0000-0003-3388-0107), Andrew J. Schaub^5^ (0000-0001-7770-7045), Jon R. Sobus^4^ (0000-0003-0740-6604)

^1^ U.S. Food and Drug Administration, Center for Food Safety and Applied Nutrition, College Park, MD 20740

^2^ University of Washington Tacoma, Center for Urban Waters, Tacoma WA 98421 (current)

^3^ National Institute of Standards and Technology, Charleston SC 29412 (former)

^4^ U.S. Environmental Protection Agency, 109 TW Alexander Dr., Research Triangle Park, NC USA 27709

^5^ Southwest Research Institute, Intelligent Systems Division, San Antonio TX 78228

*Corresponding Authors:

Christine M. Fisher; [Christine.odonnell@fda.hhs.gov](mailto:Christine.odonnell@fda.hhs.gov)

Katherine T. Peter; [ktpeter@uw.edu](mailto:ktpeter@uw.edu)

**Table of Contents**

**Text S1.** Mass Spectrometry for Targeted Analysis………………………………………..……S2

**Text S2.** Mass Spectrometry for Non-Targeted Analysis………………………………………...S2

**Text S3.** Performance Metrics Associated with the Confusion Matrix………………………….S5

**Text S1. Mass Spectrometry for Targeted Analysis**

Mass spectrometry is often utilized for targeted chemical analysis owing to its performance characteristics (regarding specificity, sensitivity, accuracy, and precision) and widespread availability. Mass spectrometers are designed and manufactured in a wealth of configurations, enabling the targeted analysis of vast organic and inorganic species. To increase performance, mass spectrometry is commonly paired with chemical separation (such as gas or liquid chromatography) that control the order and speed with which chemical species enter the mass spectrometer and become ionized and subsequently detected. The fundamental measure of a mass spectrometer is a mass-to-charge ratio, or *m/z*. In a typical MS experiment, charged molecules of a given mass are detected and reported to the user as measured *m/z* signals. With some knowledge of expected ion types (e.g., M^+^, [M+H]^+^, [M-H]^-^), MS software can easily convert an observed *m/z* signal to an estimated mass, and thus support mass-based compound identification.

Many targeted analytical methods use low-resolution mass spectrometers. These instruments scan a user-defined *m/z* range and report observed signals within that range. Any true *m/z* signal can be reported to the user with unit mass resolution (i.e., a mass [in Daltons] rounded to the nearest integer). To minimize interferences and boost true signals, targeted methods generally monitor narrow *m/z* windows (i.e., 1-Dalton intervals) that correspond to target analytes; the selection of specific *m/z* windows (and corresponding chromatographic time windows) is directed by preliminary experiments with prepared chemical standards. Even with narrowly defined windows, co-eluting chemicals (i.e., those not sufficiently separated by chromatography) can share a nominal mass, thus yielding convoluted spectra and producing challenges for qualitative and quantitative interpretation. Therefore, it is common practice to overcome these challenges using tandem MS analysis (in which target analytes are isolated and then fragmented, with *m/z* reported for observed product ions) and/or by developing intricate chromatographic separations. However, when MS data is acquired in this manner for pre-selected targets (regardless of chromatographic separations and/or MS fragmentation), *m/z* signals for all other chemicals go undetected and unreported.

**Text S2. Mass Spectrometry for Non-Targeted Analysis**

While the following information has been covered in other articles [1-3], an understanding of the fundamentals of NTA data underpins the ability to assess NTA method performance. Thus, we include background information here to provide the necessary context for NTA researchers, stakeholders, and individuals less familiar with NTA data.

Targeted analysis methods are not intended to enable discovery of new/unknown chemicals, given that they require structure-matched chemical standards (and *a priori* knowledge), and generally disregard chemical signals not associated with defined analytical targets. With the growing need to characterize a vast number of emerging contaminants, the NTA research community has turned to high-resolution mass spectrometry (HRMS) platforms, particularly those utilizing time-of-flight (ToF) or Orbitrap mass analyzers. Both low- and high-resolution mass spectrometers measure the *m/z* of molecules after ionization (MS data) and many instruments can further fragment the ionized molecules and measure the *m/z* of those fragments (MS/MS data). However, unlike low-resolution mass spectrometers, HRMS instruments measure *m/z* signals with high accuracy and resolution. Generally speaking, whereas low-resolution mass spectrometers accurately report the integer (nominal) mass of a given analyte (e.g., acetaminophen = 151 Da), high-resolution mass spectrometers accurately report mass at the tenths to the ten-thousandths of a Dalton (e.g., 151.0633 Da; typically <10 ppm mass error). (We refer readers to [4-8] for descriptions of analytical performance metrics across various HRMS platforms). This allows clear differentiation between chemical species of the same nominal mass and offers clues as to the elemental composition of any observed analyte. Further clues are then provided by isotopologue peaks (defined below) present in the precursor (MS) ion spectra.

Often (for smaller compounds with few or no halogens), the most abundant *m/z* signal in a compound-specific MS spectrum corresponds to the form of that compound containing only the most abundant stable isotope of each element (e.g., C^12^, H^1^, Cl^35^, etc.). The mass associated with this form of the compound is the “monoisotopic mass”. A calculatable percentage of molecules for any given compound will contain atoms of one or more less-abundant isotopes (e.g., C^13^, deuterium, Cl^37^, etc.). These molecules are called isotopologues and yield distinct *m/z* signals when detected via HRMS. Because the natural abundances of elemental isotopes are well established, an observed analyte’s molecular formula can often be predicted given an estimated monoisotopic mass and empirical distribution of isotopologue peaks (and vice versa). Additional information contained within product ion (MS/MS) spectra can be further used to inform the analyte’s molecular formula and structure, which is particularly useful given that it is common for multiple compounds to have the same elemental composition.

Additionally, even when introduced into “soft” HRMS ion sources (e.g., electrospray ionization [ESI] sources), compounds can partially fragment, form as multimers, or combine with other ions (e.g., sodium, potassium, ammonium) to form adducts. Each of these individual MS ions (e.g., in-source fragment, adduct, multimer, and isotopologue *m/z* peaks; each observed as a *m/z*-retention time pair, abbreviated mz@RT) represent “components” of a single compound. An important step of HRMS data processing (using either vendor-specific or open-source software) is grouping these MS components into “features” that correspond to individual compounds. Associated MS/MS product ions may also be grouped with the MS components of a feature during HRMS data processing by some software algorithms. A feature is typically reported as the observed monoisotopic mass, RT, and overall intensity (e.g., peak area or peak height) and has a corresponding composite spectrum (containing all grouped *m/z* signals and their individual intensities). Many customizable software parameters can influence the ion grouping and feature reporting. For example, designated signal intensity thresholds and peak grouping criteria can each vastly affect the final feature count. These thresholds/criteria are selected by the user for each application to best suit the study objectives and must be carefully reported.

Given the difficulty in fine-tuning feature selection settings, related *m/z* signals may not be correctly grouped for all unknowns, leading to an overreporting of features with unique, but perhaps incorrect, monoisotopic masses (e.g., an mz@RT that is actually an [M+Na]^+^ adduct of Compound A is incorrectly annotated as the [M+H]^+^ adduct of Compound B). Such errors can contribute to false positive compound identifications in downstream analyses. For a different percentage of unknowns, unrelated *m/z* signals may be incorrectly grouped, leading to one or more false negatives in downstream analyses, since at least one unknown analyte is not uniquely represented as a distinct molecular feature (e.g., an mz@RT that is actually the [M+H]^+^ adduct of Compound B is incorrectly annotated as an [M+Na]^+^ adduct of Compound A). Furthermore, an improper grouping of unrelated *m/z* signals can yield an incorrect monoisotopic mass for a single reported feature. Again, all downstream efforts towards compound identification will be affected by the initial incorrect annotation of the feature’s monoisotopic mass.

A rigorous evaluation of feature selection procedures is desirable but extraordinarily challenging given the overwhelming number of molecular features detected in any given sample (hundreds to tens-of-thousands), and the difficulty in determining the true condition of any given feature (i.e., whether *m/z* signals were correctly grouped and the monoisotopic mass correctly assigned). Recent efforts in the field of metabolomics have addressed this issue by growing bacteria in ^13^C labeled media to distinguish true features (^13^C-labeled compounds) from background features (unlabeled) [9]. While informative for biological sample analyses, these methods are not transferable to non-biological samples. Thus, in many applications, it can be challenging to assess the performance of *m/z* grouping procedures and feature extraction procedures, especially when short chromatographic gradients are used with complicated sample matrices. As a general rule, the total number of molecular features detected by a method is not necessarily a good indicator of performance. In other words, detecting more features does not necessarily indicate better chemical coverage. The detection of more features could suggest the true presence of more unique compounds or, instead, indicate a failure to remove artifact/noise/background signals and/or to correctly group adduct/fragment/isotopologue peaks. Additionally, given all the aforementioned challenges, different software platforms may yield distinct feature lists for the same data set (depending on factors such as selected settings, samples included during data processing, etc.). Thus, developing a rigorous “true” feature list (i.e., containing only non-noise features, representing all chemicals truly detected by the instrument and present in the raw data, reproducible across software platforms) currently remains difficult. In light of these challenges, a full discussion of performance metrics at the feature level was not included in this article. Nevertheless, it is critical to remember that feature selection decisions affect all downstream results, and in turn, performance metrics related to those results.

**Text S3. Performance Metrics Associated with the Confusion Matrix**

The performance metrics described below are often used interchangeably and/or in tandem, which is reflected by the grouping below. However, these groups are not exhaustive and often depend on the goals of the analysis and types of imbalances in the data (e.g., precision and recall, and True Positive Rate and True Negative Rate are two other common metric pairs). Refer to **Figure 2** in the main text for metric calculations. The information below reflects information provided on the BP4NTA website ([www.nontargetedanalysis.org](http://www.nontargetedanalysis.org)) and is not exhaustive; many further resources exist that describe the confusion matrix and associated metrics in detail (e.g., [10-12]).

**Group 1: True Positive Rate** (TPR; also known as “Recall”, “Sensitivity”, or “Hit Rate”) and **False Negative Rate** (FNR; also known as “Miss Rate”)

- - - - FNR + TPR = 1
      - For sample classification:
        - TPR = the proportion of samples that were correctly reported as the positive condition (e.g. reported *and* *actually* adulterated), relative to all samples that actually are the positive condition (e.g., actually adulterated).
        - FNR = the proportion of samples that were incorrectly reported as the negative condition (e.g., reported authentic *but* *actually* adulterated), relative to all samples that actually are the positive condition (e.g., actually adulterated).
      - For chemical identification:
        - TPR = the proportion of compounds that were correctly reported in a sample, relative to all compounds known to actually be present in the sample.
        - FNR = the proportion of compounds that were incorrectly reported as not present (i.e., not identified) in a sample, relative to all compounds known to actually be present in the sample.

**Group 2: False Positive Rate** (FPR; also known as “Fall-Out”) and **True Negative Rate** (TNR; also known as “Specificity” or “Selectivity”)

- FPR + TNR = 1
- For sample classification:
  - FPR = the proportion of samples that were incorrectly reported as the positive condition (e.g., reported adulterated *but* *actually* authentic), relative to all samples that actually are the negative condition (e.g., actually authentic).
  - TNR = the proportion of samples that were correctly reported as the negative condition (e.g., reported authentic *and actually* authentic), relative to all samples that actually are the negative condition (e.g., actually authentic).
- For chemical identification:
  - FPR = the proportion of compounds that were incorrectly reported in the sample, relative to all compounds that are actually not known to be present in the sample.
  - TNR = the proportion of compounds that were correctly reported as not present (i.e., not identified) in the sample, relative to all compounds that are actually not known to be present in the sample.
  - Use of these metrics to evaluate chemical identification performance requires caution, as the number of TNs depends on the size of the considered chemical space for the performance assessment. Disproportionately large numbers of TNs can yield artificially good TNR and FPR.

**Group 3: Precision** (“Positive Predictive Value”) and **False Discovery Rate** (FDR)

- Precision + FDR = 1
- For sample classification:
  - Precision = the proportion of samples that were correctly reported as the positive condition (e.g., reported adulterated *and* *actually* adulterated), relative to all samples reported as the positive condition (e.g., reported adulterated).
  - FDR = the proportion of samples that were incorrectly reported as the positive condition (e.g., reported adulterated, *but* actually authentic), relative to all samples reported as the positive condition (e.g., reported adulterated).
- For chemical identification:
  - Precision = the proportion of compounds correctly identified in the sample, relative to all compounds reported in the sample.
  - FDR = the proportion of compounds incorrectly identified in the sample, relative to all compounds reported in the sample.

**Group 4: Accuracy, F_1_ Score**, and **Matthew’s Correlation Coefficient (MCC)**

- Accuracy
  - For sample classification, Accuracy = the proportion of samples that were correctly reported (for both positive and negative conditions), relative to all samples.
  - For chemical identification, Accuracy = the proportion of compounds correctly reported as present or not present, relative to all compounds considered within the defined boundary of the analysis.
  - Note: Unbalanced datasets, which have more observations in one specific class than in the other(s), will yield a biased Accuracy score (or “Accuracy Paradox”). For example, consider classifying food samples as adulterated or authentic, where 1 in 100 samples is adulterated. If the method classifies 98 of the authentic samples correctly, one authentic sample as adulterated, and the adulterated sample as authentic, then the Accuracy is 98%, which suggests excellent classification performance. In reality, the method misclassified all adulterated samples. Thus, Accuracy should be combined with F_1_ Score and/or Matthew’s Correlation Coefficient (MCC; described below) to give a more well-rounded description of performance [13, 14].
- F_1_ Score
  - Calculated as the harmonic mean of TPR and Precision; thus, perfect Recall and Precision will yield F_1_ score = 1.
  - F_1_ Score, like TPR and Precision, does not include the number of true negatives. Therefore, the choice of the positive condition (e.g., adulterated food vs. authentic food) can influence F_1_ score. Convention dictates that the rarer or more interesting class (e.g., adulterated food, relative to authentic food) will be the positive condition.
- Matthew’s Correlation Coefficient (MCC)[14, 15]
  - MCC uses all values in the confusion matrix, and ranges from -1 (perfect misclassification) to 1 (perfect classification), with values near zero indicating a random guess.
  - MCC is useful for unbalanced data sets, which have more observations in one specific class than in the other(s). Likewise, the invariant nature of MCC allows comparison of NTA studies that used different numbers of samples. MCC treats each class as a variable and computes their correlation coefficient.
  - For example, MCC has been used, along with Accuracy, in metabolomics studies to evaluate performance in the detection of biomarkers [16] and in the classification of different liver diseases in children [17].

**References**

1. Schymanski EL, Jeon J, Gulde R, Fenner K, Ruff M, Singer HP, et al. Identifying small molecules via high resolution mass spectrometry: Communicating confidence. Environmental Science and Technology. 2014;48(4):2097-8.

2. Hollender J, Schymanski EL, Singer HP, Ferguson PL. Nontarget screening with high resolution mass spectrometry in the environment: Ready to go? Environmental Science and Technology. 2017;51(20):11505-12.

3. Fisher CM, Croley TR, Knolhoff AM. Data processing strategies for non-targeted analysis of foods using liquid chromatography/high-resolution mass spectrometry. TRAC Trends in Analytical Chemistry. 2021;136:116188.

4. Kaufmann A, Teale P. Chemical analysis of non-antimicrobial veterinary drug residues in food. In: Kay JF, MacNeil JD, Wang J, editors. Capabilities and limitations of high-resolution mass spectrometry (HRMS): Time-of-flight and orbitrap. 2016. p. 93-139.

5. Kaufmann A. Combining UHPLC and high-resolution MS: A viable approach for the analysis of complex samples? TRAC Trends in Analytical Chemistry. 2014;63:113-28.

6. Zubarev RA, Makarov A. Orbitrap mass spectrometry. Analytical Chemistry. 2013;85(11):5288-96.

7. Xian F, Hendrickson CL, Marshall AG. High resolution mass spectrometry. Analytical Chemistry. 2012;84(2):708-19.

8. Knolhoff AM, Callahan JH, Croley TR. Mass accuracy and isotopic abundance measurements for HR-MS instrumentation: Capabilities for non-targeted analyses. Journal of the American Society for Mass Spectrometry. 2014;25(7):1285-94.

9. Mahieu NG, Patti GJ. Systems-level annotation of metabolomics data reduces 25,000 features to fewer than 1,000 unique metabolites. Analytical Chemistry. 2017;89(19):10397-406.

10. Fawcett T. An introduction to ROC analysis. Pattern Recognition Letters. 2006;27(8):861-74.

11. Powers DM. Evaluation: From precision, recall and f-factor to ROC, informedness, markedness & correlation. International Journal of Machine Learning Technology. 2011;2(1):37-63.

12. Luque A, Carrasco A, Martín A, de las Heras A. The impact of class imbalance in classification performance metrics based on the binary confusion matrix. Pattern Recognition. 2019;91:216-31.

13. Chicco D, Jurman G. The advantages of the matthews correlation coefficient (MCC) over F1 score and accuracy in binary classification evaluation. BMC Genomics. 2020;21(1):6.

14. Baldi P, Brunak S, Chauvin Y, Andersen CA, Nielsen H. Assessing the accuracy of prediction algorithms for classification: An overview. Bioinformatics. 2000;16(5):412-24.

15. Matthews BW. Comparison of the predicted and observed secondary structure of t4 phage lysozyme. Biochimica et Biophysica Acta. 1975;405(2):442-51.

16. Depner CM, Cogswell DT, Bisesi PJ, Markwald RR, Cruickshank-Quinn C, Quinn K, et al. Developing preliminary blood metabolomics-based biomarkers of insufficient sleep in humans. Sleep. 2020;43(7).

17. Kordy K, Li F, Lee DJ, Kinchen JM, Jew MH, La Rocque ME, et al. Metabolomic predictors of non-alcoholic steatohepatitis and advanced fibrosis in children. Frontiers in Microbiology. 2021;12(2275):713234.
